# Structure and Process of Infrared Hot Electron Transistor Arrays

**DOI:** 10.3390/s120506508

**Published:** 2012-05-16

**Authors:** Richard Fu

**Affiliations:** U.S. Army Research Laboratory, 2800 Powder Mill Road, Adelphi, MD 20783, USA; E-Mail: richard.x.fu.civ@mail.mil; Tel.: +1-301-394-1473; Fax: +1-301-394-0310

**Keywords:** quantum well infrared photodetector (QWIP), infrared hot-electron transistor (IHET), GaAs

## Abstract

An infrared hot-electron transistor (IHET) 5 × 8 array with a common base configuration that allows two-terminal readout integration was investigated and fabricated for the first time. The IHET structure provides a maximum factor of six in improvement in the photocurrent to dark current ratio compared to the basic quantum well infrared photodetector (QWIP), and hence it improved the array S/N ratio by the same factor. The study also showed for the first time that there is no electrical cross-talk among individual detectors, even though they share the same emitter and base contacts. Thus, the IHET structure is compatible with existing electronic readout circuits for photoconductors in producing sensitive focal plane arrays.

## 1. Introduction

There is an urgent need for affordable, sensitive and high resolution long wavelength (λ ∼ 10 μm) infrared focal plane arrays (LWIR FPAs) for large area persistent surveillance. A LWIR camera with resolution of one million pixels (MP) or more is required [1]. The conventional LWIR FPAs are expensive and the resolution is limited to 0.3 MP. Quantum well infrared photodetector (QWIP) FPAs are less expensive and potentially offer higher resolutions. We recently demonstrated several 1 MP corrugated-QWIP LWIR FPA cameras, which show great promise for this application [2]. To further improve the technology, it is important to increase the detector sensitivity and photocurrent to dark current ratio of the FPAs. In the very long wavelength (VLWIR, λ ∼ 14 μm) regime, the dark current problem is further exacerbated by their lower barrier heights, in which the dark current is not only contributed by the TAT current but also by the thermionic emission (TE) current conducting above the structural barriers [3]. To improve the detector sensitivity, the dark current from different transport mechanisms has to be suppressed.

The objective of this work is to demonstrate an advanced QWIP sensor in a small exploratory array format, which is capable of suppressing the detector dark current. The detector is known as the infrared hot-electron transistor (IHET) [4]. An IHET is a quantum well infrared photodetector (QWIP) with a built-in electron energy filter and contains three terminals. The QWIP is located between the emitter and base terminals, and the filter is located between the base and collector terminals. The emitter and base are used to supply an operating voltage to the QWIP. The filter is used to accept photoelectrons of certain energies into the collector and rejects the dark electrons from other energies. The rejected electrons will then drain through the base terminal. By accepting only electrons at particular energies, the filter reduces the dark current and increases the photocurrent to dark current ratio at the collector. Consequently, the sensitivity of the detector can be increased. In this work, we designed the detector structure and characterized the potential performance enhancements, and fabricated small format (5 × 8) IHET detector arrays for the first time to ascertain their advantages.

IHETs in discrete form had been studied in the past and the expected detector functionality had been observed [5–8]. However, they have not been demonstrated in array formats due to the special contact requirements. Specifically, each IHET requires three external contacts for detector operation, while the usual QWIPs need only two. The existing readout integrated circuits (ROICs) were designed for two-terminal detectors, in which there is only one individual top contact per pixel. A unique array architecture has to be devised for the IHETs if one is to utilize the existing ROICs. In this work, we propose an architecture that will require only one individual contact for each IHET, and thus it is more compatible with the existing ROICs. In this array format, the collector of the IHET is contacted individually while the emitter and the base are both common to all pixels. It will require the active QWIP layers of all pixels in an array column be joined together. This is feasible only if the current in each pixel is flowing strictly perpendicular to the layers. In principle, this will be the case because the electric field between the emitter and the base is perpendicular to the layers. It is prudent however to measure any lateral diffusion of carriers into the neighboring pixels thus burring an optical image. Another purpose of this work is to determine the electrical cross-talk among different pixels.

## 2. Approach

**Figure 1.**
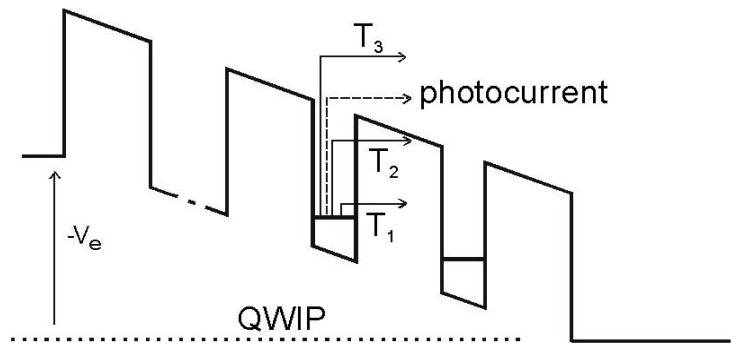
The band diagram of a typical QWIP and the energies of the dark current and photocurrent at different temperatures.

**Figure 2.**
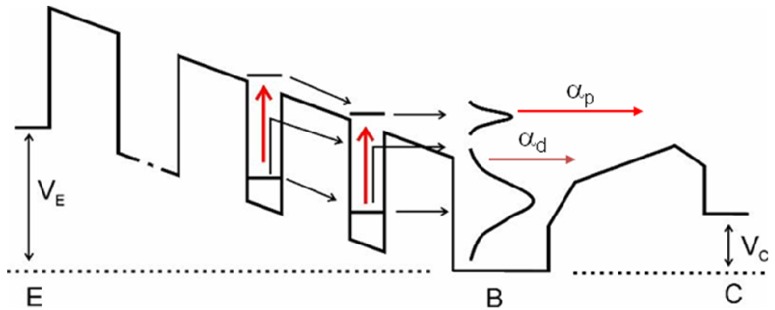
The IHET structure with a thick barrier near the collector C as a high pass filter. In this illustration, the higher energy photoelectrons created by optical transition (red arrows) is accepted into the collector while the lower energy TAT current is rejected into the base.

To understand the advantages of an IHET, we need to look into the conduction mechanisms of a typical QWIP. For this purpose, we show the QWIP energy band diagram under a bias V_E_ in Figure 1. At a low operating temperature (T), such as at T_1_, direct tunneling current is responsible for the dark current flow. At a moderate temperature of T_2_, thermally assisted tunneling (TAT) below the barriers is more significant, and at a high temperature of T_3_, thermionic emission above the barriers dominates. Consequently, the energy of the predominant current flow depends on T. On the other hand, the energy of the photoelectrons depends only on the incoming photon energy and is independent of T. In most T, the energy of the photoelectrons is different from that of the dark electrons, and thus they can be separated by an energy selective filter. By attaching an additional barrier at the end of the anode (the base) as in Figure 2, one can regulate the passage of the electrons into the new collector terminal according to their energies. With an appropriate collector bias, one can maximize the photocurrent to dark current ratio.

**Figure 3.**
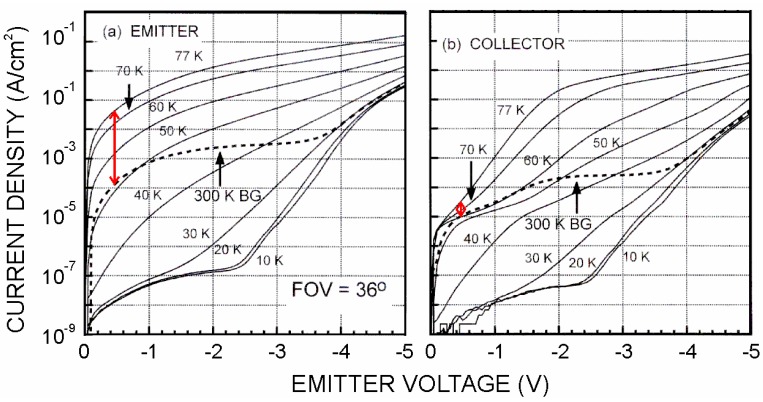
(**a**) The dark current and photocurrent measured at the emitter before filtering. (**b**) The dark current and the photocurrent after filtering.

Figure 3 shows the I-V characteristics of an IHET detector with a double barrier filter, which acts a bandpass filter. It rejects the electrons both below and above the pass band. When the pass band is tuned to align with the photoelectron energy, a high degree of dark current rejection will be achieved. For example, before filtering, the emitter dark current shown in Figure 3(a) is 8 × 10^−2^ A/cm^2^ at V_E_ = −0.5 V and T = 77 K, which is 400 times larger than 300 K blackbody background photocurrent. After filtering, the collector dark current in Figure 3(b) is only 5 times higher. This example shows that an IHET structure can substantially reduce the dark current and improves the operating temperature and the sensitivity of a QWIP [5].

**Figure 4.**
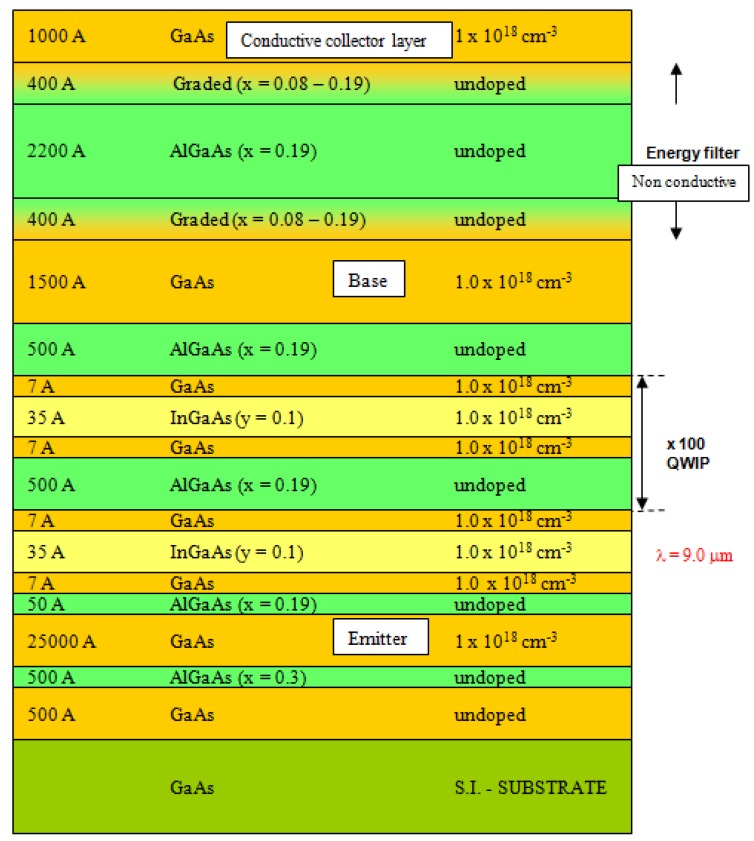
Layers structure of IHET design.

Figure 4 is the layers structure of the IHET design. One emitter layer, multi-QWIP layers and one base layer are grown on GaAs substrate; an energy selective filter is located between the base and collector terminals. The epitaxial layers studied in the present work were grown on 4 inches GaAs (100) substrates by a molecular beam epitaxy. Prior to the growth of the full In_x_Ga_1−x_As/In_y_Ga_1−y_As IHET structure which is almost 9 μm thick, an In_x_Ga_1−x_As/GaAs quantum well (QW) superlattice (SL), an In_y_Ga_1−y_As/bulk layer, and mini-IHET (2 μm thick) were grown and characterized for the optimized growth condition calibration. The mini-IHET is a shortened version of the full structure IHET; 11 multi-QW (MQW) periods instead of 100. Silicon was the donor dopant with nominal concentration of 1 × 10^18^ cm^−3^ in the emitter layer, base layer, collector layer, and in the InGaAs wells, respectively. The MQW structure was sandwiched between a heavily doped GaAs layer (2.5 μm thick) as the emitter contact and a heavily doped GaAs layer as the base layer (150 nm). On top of the base, there was an undoped AlGaAs filter layer (220 nm) followed by a heavily doped GaAs layer (100 nm) as the collector.

**Figure 5.**
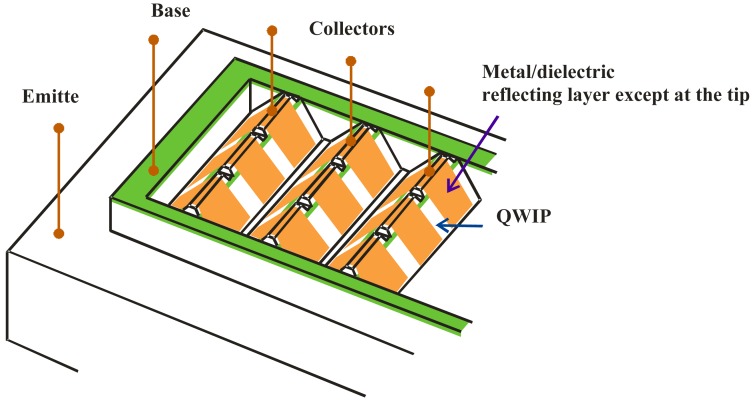
The schematic drawing of an IHET array with common emitter and base contacts.

Figure 5 shows the schematic of an IHET array. In this design, both the emitter and the base contacts are common to all pixels. The two contacts set the potential across the QWIP detectors. The collector contact is made individually to each detector pixel. The metal/dielectric cover reflects the normal incident light into parallel propagation as needed for QWIP absorption. The active QWIP layers of different pixels are connected in each column of the array. The present connection arrangement is to avoid having two individual contacts for each pixel and thus achieves a higher packing density and is more compatible with the existing readout circuits.

**Figure 6.**
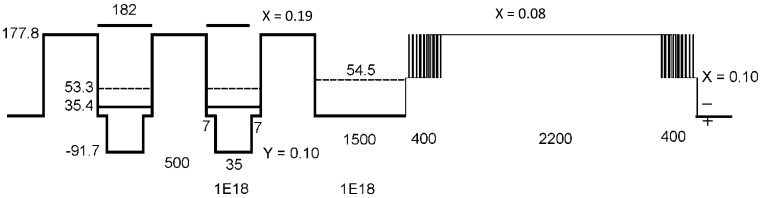
The band diagram and the structural parameters of the present IHET design.

The IHET structure used in the present project is shown in Figure 6. It is designed to have a 9.2 μm wavelength cutoff. Calculated conduction band-edge profile at zero bias shows a two QWIP period with the structural parameters (x, y, well width, barrier width) of the In_y_Ga_1−y_As/GaAs/Al_x_Ga_1−x_As IHET design.

## 3. Experimental Section

**Figure 7.**
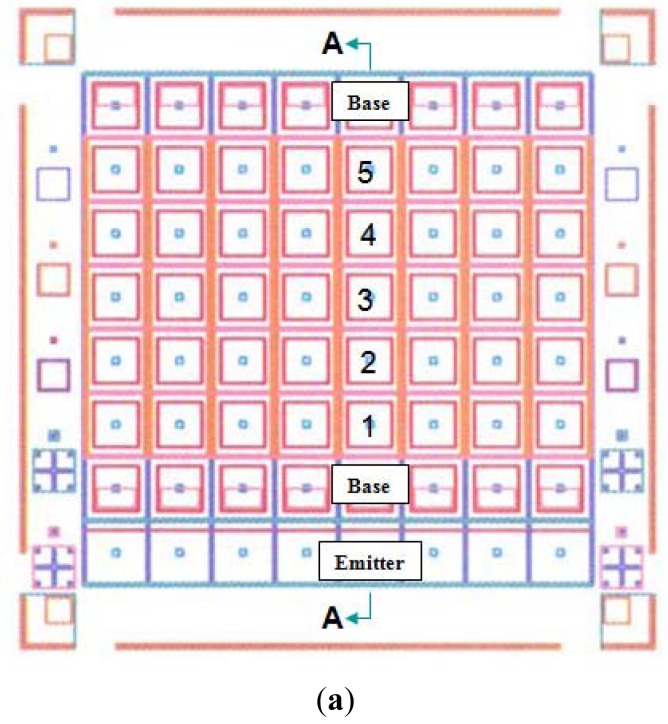

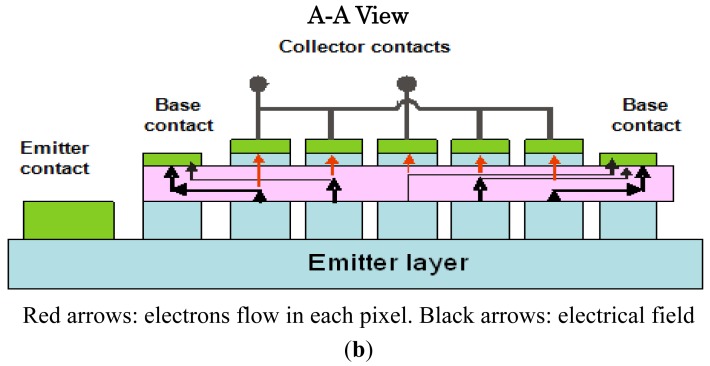
(**a**) The mask layout of the IHET array. (**b**) The cross-section of an array column.

We investigated the common base configuration shown in Figure 5. This approach is to eliminate the need to have two external contacts for each detector, one for the base and another for the collector. Figure 7(a) shows the mask layout of a 5 × 8 IHET array. The bottom row is the emitter layer contact common to all pixels. The adjacent row and the top row are the base contacts common to individual detector columns. The center five rows are individual detector collector contacts. To properly operate each array column, a negative V_E_ is applied to emitter contact, a ground contact is connected to the two base contacts and a small voltage is applied to individual collector contacts. Figure 7(b) shows the cross-section on an array column.

**Figure 8.**
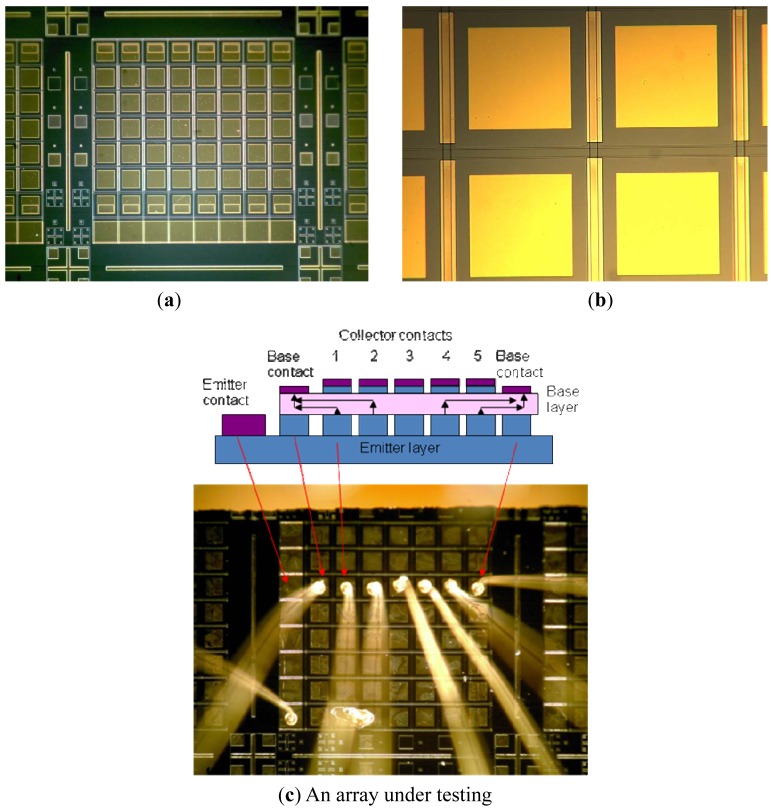
Processed IHET arrays. (**a**) Processed arrays; (**b**) Individual detectors.

To fabricate this detector architecture, mixture of phosphoric acid, hydrogen peroxide and water (H_3_PO_4_):(H_2_O_2_):(H_2_O) in 1:1:3 proportion was chosen to etch to emitter GaAs layer and divided the wafer into array columns in V shapes. The plasma dry etching process was conducted in a Unaxis VLR 700 etching system. The etcher has a helium backside cooling configuration and a mechanical ceramic clamp to hold the wafer. The photoresist was patterned on top of the wafer to act as an etching mask. Plasma dry etching parameters were applied as follows: gas flows: BCl_3_ = 25 sccm, Ar = 5 sccm; chamber pressure = 2.5 mTorr; helium pressure = 4,000 Psi; RF1 (RIE) power = 150 W; RF 2 (ICP) power = 600 W; and substrate temperature = 25 °C. The etching depth was measured by a Tencor P-15 Profilometer. Precision shallow plasma etching was used to reach vertically down to the base layer and divided the array into rows. Another deep plasmas etching was to separate the emitter from the base in the same column as shown in Figure 7(b). Shallow ohmic contacts consisting of 15 nm Pd, 20 nm Ge and 200 nm Au were deposited on all contacts by e-beam evaporator and were alloyed at 420 °C for 100 seconds using rapid thermal annealing (RTA). Finally, 200 nm MgF_2_, 10 nm Cr and 200 nm Au were deposited on the detector sidewalls as optical reflectors by e-beam evaporator. The arrays were diced and polished with a 45° facet for optical coupling. For array testing, a row of detectors were wire bonded and mounted in an optical Dewar in Figure 8(c).

Figure 8 shows the processed whole IHET 5 × 8 arrays (a), individual detectors (b) and a row of wire bonded detectors under testing. The individual detector size is 25 μm × 25 μm, the pixel pitch is 25 μm.

## 4. Results and Discussion

### 4.1. Photocurrent vs. Dark Current

**Figure 9.**
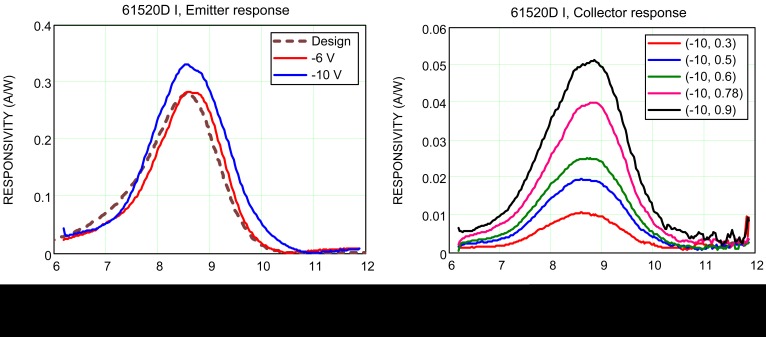
(**a**) Emitter spectral response at V_E_ = −6 and −10 V. (**b**) Collector response at V_E_ = −10 V and V_C_ = 0.3 – 0.9 V.

**Figure 10.**
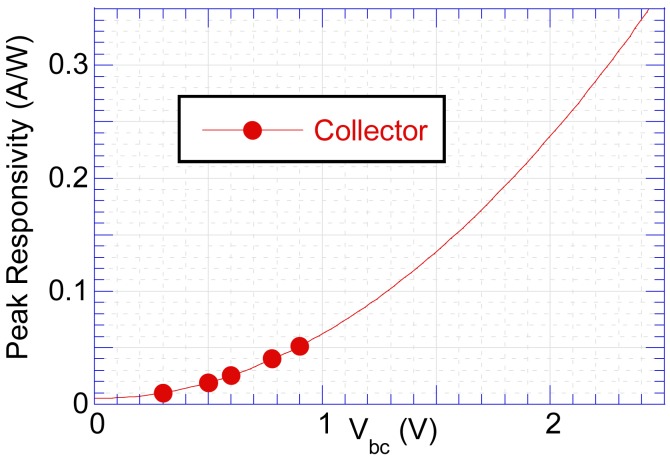
Extrapolating relationship between peak responsivity and V_C_.

Emitter and collector spectral responses from a test array were measured at different V_E_ and V_C_. The results are shown in Figure 9. The emitter responsivity, which represents the response from the QWIP, matches the designed spectrum, confirming the QWIP material growth. The collector response R_C_ is however about one order of magnitude less than the emitter response R_E_, showing that there is a large photocurrent reduction. Extrapolating the trend of R_C_ *vs.* V_C_ in Figure 10, it would require a large V_C_ of 2.5 V to capture ∼80% of the photoelectrons.

**Figure 11.**
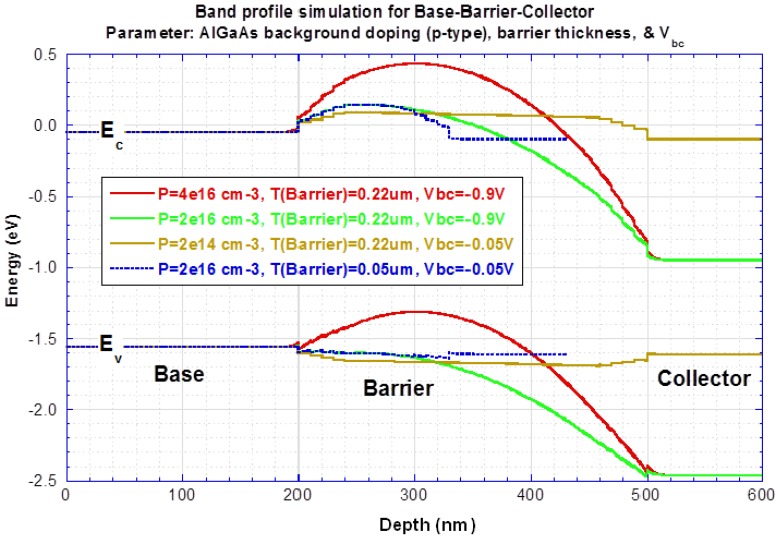
Barrier height *vs.* p-type background doping.

**Figure 12.**
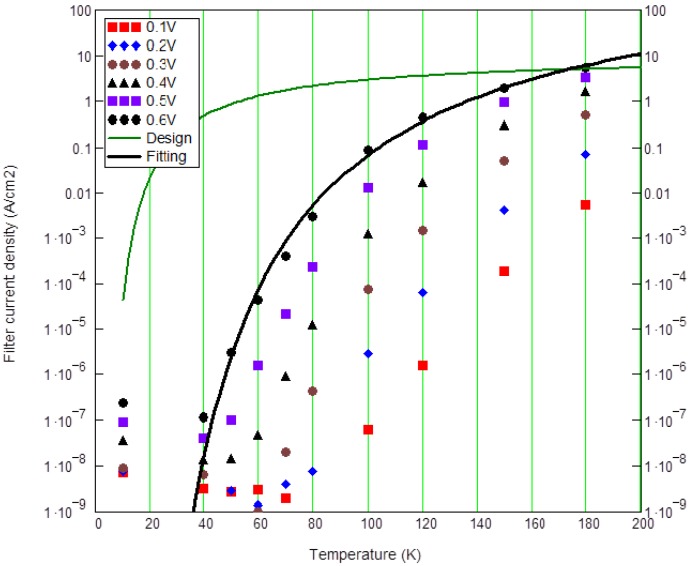
Filter current density *vs.* temperature.

**Figure 13.**
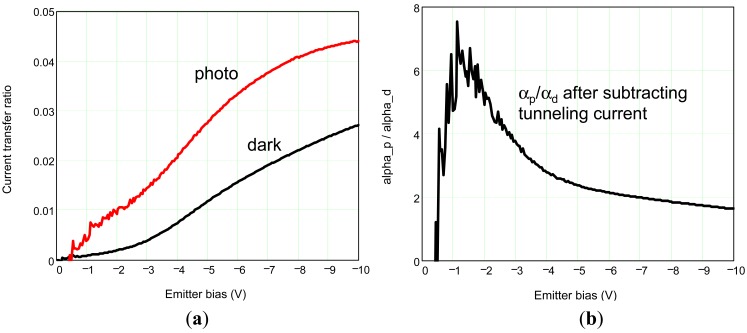
Emitter to collector current transfer ratio curves *vs.* emitter bias at V_C_ = 0.4 V.

Suppressing dark current depends on the energy level and barrier thickness (or barrier height) of the filter. The apparent barrier height of the filter in the present material was higher than the designed value. This large V_C_ requirement is attributed to the finite p-type background doping in the filter barrier that raises the filter barrier height in Figure 11 (Red: actual measurement, −0.9 V; Orange: ideal design, −0.05 V; Green: lower doping, but −0.9 V too high; Blue: thinner and lower doping, −0.05 V). Based on Figure 6, the designed activation energy is 63.3 meV (177.8 – 54.5). From actual fitting results in Figure 12, large fitted activation energy is 1,023 meV at V_c_ = 0.6 V, which is 16 times higher than the designed (63.3 meV) barrier height. The p-type doping not only raises the barrier height but also places the highest point of the barrier in the middle of the IHET layer structure instead in the front. This increases the possibility of the photoelectrons to lose their energy before being selected. The higher actual activation energy and the longer photoelectron traveling path make the large photocurrent reduction. But the dark current transfer ratio α_d_ is even lowered by the same p-doping. Therefore, we are still collecting more photoelectrons than dark electrons in Figure 13.

Figure 13(a) compares the dark current transfer ratio α_d_ at 77 K (black curves) and the photocurrent transfer ratio α_p_ (red curves) at V_C_ = 0.4 V. It is apparent that α_p_ > α_d_ in the entire bias range. The largest α_p_/α_d_ is 6 at V_E_ = −1.2 V as shown in Figure 13(b). The filtering factor should be improved by systematically studying on optimization of p-type doping with material growth partners to decrease barrier height.

### 4.2. Detector Electrical Cross-Talk

**Figure 14.**
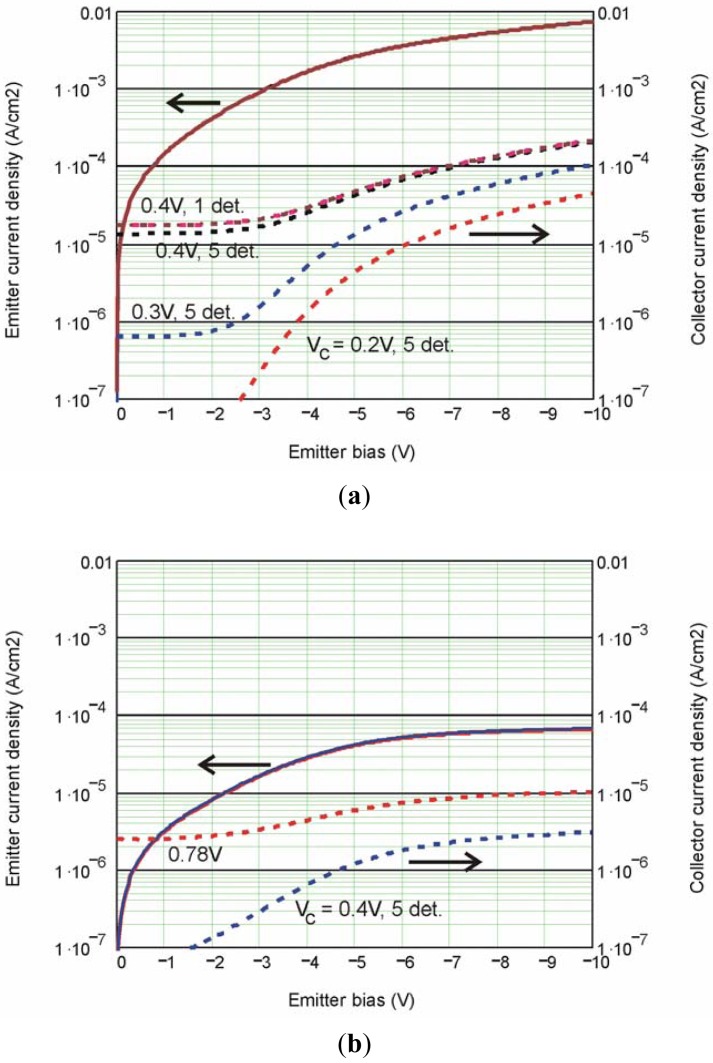
(**a**) Dark current density and (**b**) Photocurrent density *vs.* V_E_ at 77 K.

The emitter and collector dark current data are shown in Figure 14(a). The overlapping solid curves on top are the emitter current density, J_E_, at different V_C_ and different number of collectors being connected. This constant J_E_ indicates good electrical isolation between the QWIP stage and the collector stage. The dashed curves are the collector current densities J_C_. The large reduction of dark current is also consistent with a higher filter barrier. The top two overlapping dash curves are J_C_ from a single detector with the other collector contacts connected to each other or disconnected from the circuit. This excellent overlap indicates no detector cross-talk existed in this 5 × 8 array (25 μm pixel size). This important result validates that no electrical signal will transfer from one detector to the next through the common base contact. The other dash curves are J_C_ at V_C_ = 0.4, 0.3, and 0.2 V, respectively. Similarly, the photocurrent density is measured and is shown in Figure 14(b).

No electrical cross-talk among different pixels is the first time evidence that the current in each pixel is flowing strictly perpendicular to the emitter and base layers (red arrows in Figure 7(b)) because the electric field between the emitter and the base is perpendicular to the layers. It is also the first time to fabricate 5 × 8 array format that each IHET pixel (collector) is contacted individually while the emitter and the base are both common to all pixels. This two-terminal architecture makes it possible to use the existing readout integrated circuits (ROICs).

## 4. Conclusions

In summary, a 5 × 8 IHET array with a common base configuration was investigated. The IHET structure provides a maximum factor of six in improvement in the photocurrent to dark current ratio, and hence it will improve the array S/N ratio by the same factor. The study also shows that there is no electrical cross-talk among individual detectors, even though they share the same emitter and base contacts. It thus paves the way to fabricate high density sensitive focal plane arrays. To increase the photocurrent to dark current ratio and operation temperature in the future, we are going to design thinner filter barriers and improve p-type doping material growth conditions.
